# Metabolite Variations during the First Weeks of Growth of Immature *Citrus sinensis* and *Citrus reticulata* by Untargeted Liquid Chromatography–Mass Spectrometry/Mass Spectrometry Metabolomics

**DOI:** 10.3390/molecules29163718

**Published:** 2024-08-06

**Authors:** Estelle Deschamps, Marie Durand-Hulak, Denis Castagnos, Marie Hubert-Roux, Isabelle Schmitz, Yann Froelicher, Carlos Afonso

**Affiliations:** 1 Institut National des Sciences Appliquées (INSA) Rouen Normandie, Univ Rouen Normandie, Centre National de la Recherche Scientifique (CNRS), Normandie Univ, Chimie Organique et Bioorganique Réactivité et Analyse (COBRA) UMR 6014, INC3M FR 3038, 76000 Rouen, France; estelle.deschamps1@univ-rouen.fr (E.D.); marie.hubert@univ-rouen.fr (M.H.-R.); 2 EARL DURAND Olivier, Domaine de la Triballe, 34820 Guzargues, France; marie-durand@live.fr; 3 Centre de Coopération Internationale en Recherche Agronomique pour le Développement (CIRAD), UMR AGAP Institut, Station INRAE, 20230 San Giuliano, France; yann.froelicher@inrae.fr; 4 ORIL Industrie, Servier Group, 13 r Auguste Desgenétais, 76210 Bolbec, France; denis.castagnos@servier.com

**Keywords:** immature, *Citrus sinensis*, orange, *Citrus reticulata*, mandarin, metabolomics, Orbitrap, LC-MS/MS, variety discrimination, fruit development

## Abstract

Immature citruses are an important resource for the pharmaceutical industry due to their high levels of metabolites with health benefits. In this study, we used untargeted liquid chromatography–mass spectrometry (LC-MS) metabolomics to investigate the changes associated with fruit size in immature citrus fruits in the first weeks of growth. Three orange cultivars (*Citrus sinensis* ‘Navel’, *Citrus sinensis* ‘Valencia’, and *Citrus sinensis* ‘Valencia Late’) and a mandarin (*Citrus reticulata Blanco* ‘Fremont’) were separated into eight fruit sizes, extracted, and analyzed. Statistical analyses revealed a distinct separation between the mandarin and the oranges based on 56 metabolites, with an additional separation between the ‘Navel’ orange and the ‘Valencia’ and ‘Valencia Late’ oranges based on 21 metabolites. Then, metabolites that evolved significantly with fruit size growth were identified, including 40 up-regulated and 31 down-regulated metabolites. This study provides new insights into the metabolite modifications of immature *Citrus sinensis* and *Citrus reticulata* in the first weeks of growth and emphasizes the significance of including early sampled fruits in citrus maturation studies.

## 1. Introduction

Citrus fruits are well known for their nutritional properties and health benefits such as anti-inflammatory, antioxidant, anti-obesity, and anticancer properties [1]. In particular, two of the most consumed varieties, the sweet orange *Citrus sinensis* and the mandarin *Citrus reticulata*, have shown high levels of metabolites related to these health benefits, especially flavonoids and polymethoxyflavones (PMF) [2,3].

During maturation, the levels of flavonoids and PMF increase in the first months but highly decrease when reaching full maturity, while sugar levels and other metabolites increase [1,4]. Furthermore, while immature citrus fruits can be naturally harvested thanks to the phenomenon of physiological dropping [5], they are regarded as a by-product for the food industry. Conversely, they are considered a valuable source of bioactive compounds for the pharmaceutical industry. Hence, while mature citruses are appreciated for consumption, citrus fruits harvested before maturation, i.e., immature citruses, are of particular interest for the pharmaceutical industry [6,7].

Liquid chromatography (LC) coupled to tandem mass spectrometry (MS/MS) is a widely used technique for the identification and/or quantification of natural products. In recent years, untargeted LC-MS/MS metabolomics has been shown to be a powerful tool to highlight the impact of citrus varieties, growth location, maturity, or harvesting conditions on the metabolite composition of citruses. Wang et al. [8] characterized the metabolic diversity of 62 citrus varieties among sweet oranges, lemons, pummelos, and grapefruits. Sweet oranges had the highest number of flavonoid compounds, while pummelos and grapefruit had the lowest. Moreover, the authors confirmed that from all fruit tissues, the flavedo had the highest levels of flavonoids and PMF, as described elsewhere for other varieties [9,10]. Tsujimoto et al. [11] observed the discrimination of citrus-type crude drugs according to the sugar chain of flavanone glycosides (e.g., naringin vs. isonaringin). Feng et al. [12] showed that the grove location impacted the metabolite composition of *C. reticulata*. Metabolite level increases (e.g., sugars, amino acids, carotenoids) or decreases (e.g., flavonoids, lipids) were highlighted during the growth of *Citrus unshiu* [13], *C. reticulata* × *C. sinensis* [14], and *Citrus wilsonii* Tanaka [15]. Wang et al. [16] showed that infection by *Candidatus Liberibacter* sp. influenced the metabolite regulation of *C. reticulata* cv. ‘Shatangju’. However, although numerous, most metabolomic studies are performed on mature fruits. Two studies were performed by LC-UV on immature citrus at different sizes for *C. reticulata* Blanco [17] and *C. sinensis* L. Osbeck [18]. However, they were limited to four flavonoids. Since hundreds of metabolites were identified in mature citrus metabolomics studies, a much higher number of metabolites is to be expected in immature citruses.

Herein, we performed untargeted LC-MS/MS metabolomics on eight fruit sizes for four immature citrus varieties (*Citrus sinensis* ‘Navel’, *Citrus sinensis* ‘Valencia’, *Citrus sinensis* ‘Valencia Late’, and *Citrus reticulata* Blanco ‘Fremont’) collected during the first weeks of growth. Statistical analyses were then performed to determine the metabolites distinctive of the variety and fruit size.

## 2. Results and Discussion

### 2.1. Key Metabolites Responsible for Differentiating Immature Varieties

**Figure 1:**
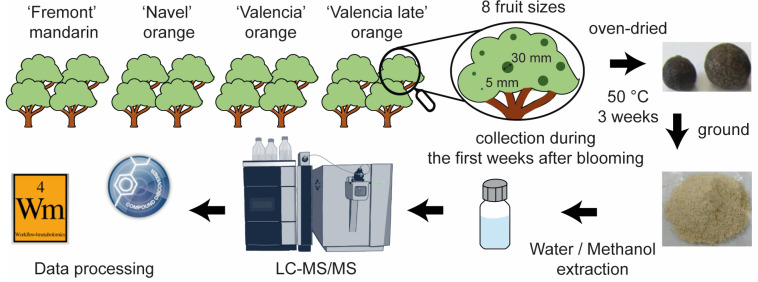
Summary diagram of the citrus metabolite analysis, from the fruit collection to the data processing.

The metabolite extracts of the immature ‘Fremont’ mandarin and the three cultivars of immature oranges (‘Navel’, ‘Valencia’, and ‘Valencia Late’) separated into eight fruit sizes per variety, with four biological replicates (128 samples in total), were analyzed by LC-MS as per recommended for metabolomics experiments [19]. Then, samples and standards were analyzed by LC-MS/MS in ddMS^2^ mode for structural elucidation. Figure 1 summarizes the whole experiment. First, data processing was carried out on all the samples. After the compound detection, QC correction, and normalization processes, the obtained matrix containing 193 RT-*m*/*z* variables, along with their corresponding normalized areas, was utilized for statistical analyses. Statistical analyses were performed to (i) compare metabolite intensity differences between varieties including all fruit sizes and (ii) determine the metabolites whose levels increased or decreased with the fruit growth. The sorted variables were then identified or putatively identified according to the annotation confidence of Schymanski et al. [20]. Examples of the three annotation levels used in this study are given in Figures S1–S4 of the Supplementary Materials.

To determine the metabolites responsible for the differentiation of varieties for each fruit size, partial least squares regression discriminant analysis (PLS-DA) was performed using the sample variety for the model. The model was statistically acceptable in terms of goodness of fit (R2X = 0.748; R2Y = 0.884) and predictability (Q2Y = 0.86).

**Figure 2:**
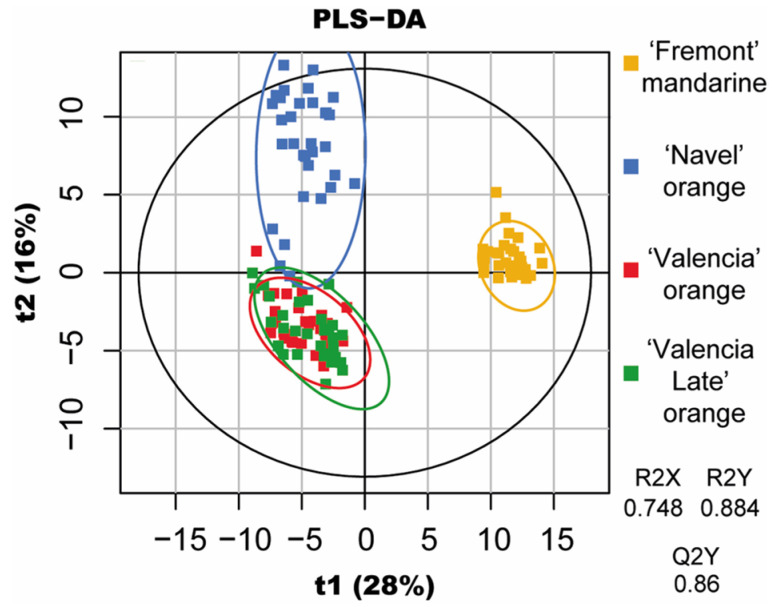
Multivariate analysis performed on all samples: PLS-DA modeled with the sample variety. Orange: Fremont mandarin, blue: ‘Navel’ orange, red: ‘Valencia’ orange, green: ‘Valencia Late’ orange.

The PLS-DA score plot was used to visualize the variety discrepancies of the immature citruses (Figure 2). The orange varieties (‘Navel’ orange, ‘Valencia’ orange, and ‘Valencia Late’ orange) were separated from the ‘Fremont’ mandarin along the PC1 axis, while the ‘Navel’ orange was separated from the ‘Valencia’ and ‘Valencia Late’ oranges along the PC2 axis. Hence, *C. reticulata* (oranges) and *C. sinensis* (mandarin) varieties were clearly separated, whereas the closest cultivars (‘Valencia’ orange and ‘Valencia Late’ orange) were not. This separation mirrors the results of Ronnigen et al. [21] on mature citruses, where PC1 separated the tangerine from the oranges and PC2 separated the ‘Valencia’ orange from the ‘Navel’ orange. In addition, the PLS-DA analysis performed using the fruit size for the models did not yield satisfactory results. This suggests that the metabolite levels were more differentiated by the variety of the fruit rather than its growth, supporting the findings of Multari et al. [22] on phenolic compounds from various citruses.

To identify the key factors that distinguish each orange cultivar from the mandarin fruit, volcano plots and PLS-DA analyses were conducted consecutively. Using the variable importance in the projection (VIP) obtained from the PLS-DA, log(fold change), and *p*-value obtained from the volcano plots, 56 variables were selected as significantly different (VIP > 1.0; log(fold change) > 1.0; *p*-value < 0.05). Among these 56 variables, 6 were identified by the corresponding standard (3′,4′,5,7-tetramethoxyflavone, sinensetin, tangeretin, 5-demethylnobiletin, nobiletin, and gardenin A), 17 were tentatively identified by correspondence with MS/MS databases, and 33 were annotated as isomers of identified compounds or by their chemical family. Details of the annotations are given in Table S2 of the Supplementary Materials.

The identified compounds could be divided into two groups: (i) up-regulated in the Fremont mandarin (18) or (ii) up-regulated in the oranges (‘Valencia’ orange, ‘Valencia Late’ orange, and ‘Navel’ orange; 38). The distribution of the identified compounds per chemical family from groups (i) and (ii) is given in Table 1.

A total of 16 flavonoids were detected in the oranges, whereas 13 were identified in the mandarins, corroborating the results of Wang et al. on mature citruses [8], which showed that sweet oranges had the highest number of flavonoid compounds among sweet oranges, mandarins, lemons, pummelos, and grapefruit. Furthermore, upon comparing the combined polymethoxyflavone intensities, the ‘Fremont’ mandarin exhibited greater levels in total PMFs compared to the three cultivars of oranges, which also concurred with the results of Wang et al. [8] and Xing et al. [23] on mature citruses.

**Figure 3:**
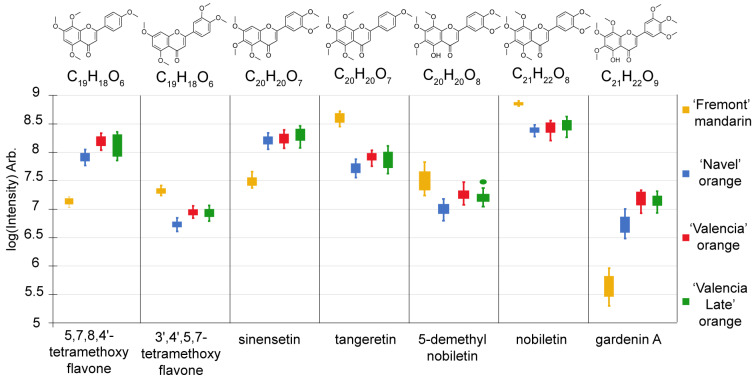
Box-plot comparison of the intensities of 6 compounds identified by the corresponding standard compounds and 1 tentatively identified compound (5,7,8,4′-tetramethoxyflavone) according to variety. Orange: ‘Fremont’ mandarin, blue: ‘Navel’ orange, red: ‘Valencia’ orange, green: ‘Valencia Late’ orange.

Figure 3 shows the comparison by boxplots of seven PMFs according to variety. Some compounds were up-regulated in oranges (e.g., 5,7,8,4′-tetramethoxyflavone, sinensetin), while their isomers were up-regulated in the mandarine (e.g., 3′,4′,5,7-tetramethoxyflavone, tangeretin). The observed sinensetin/tangeretin trend is consistent with the literature on mature *C. sinensis* [24,25,26] and *C. reticulata* [9] and in early immature fruits [25].

Although the ‘Navel’, ‘Valencia’, and ‘Valencia Late’ oranges share a close genetic relationship, a differentiation was observed in terms of metabolites between the ‘Navel’ orange and the ‘Valencia’ and ‘Valencia Late’ oranges.

To identify these metabolites, the previous methodology was repeated, leading to the selection of twenty-one significantly different variables, among which, one was identified by the corresponding standard (gardenin A) and seven were tentatively identified by correspondence with MS/MS databases. Nine compounds were up-regulated in the ‘Navel’ orange: five PMFs, one polyphenol, one limonoid, one amino sugar, and one organic acid. Twelve compounds were up-regulated in the ‘Valencia’ and ‘Valencia Late’ oranges: four PMFs, two flavones, two amino acids, one polyphenol, one amine, one amino sugar, and one lipid. Details of the annotations are given in Table S2 of the Supplementary Materials.

For both comparisons, the class of polymethoxyflavones was the most prevalent with a total of 21 compounds, including 14 hydroxylated-PMFs. Hydroxylated-PMFs are PMFs that have lost one or more methyl group, enhancing their solubility in water, albeit decreasing their capacity to penetrate biological membranes. Hence, while PMFs are better absorbed into the bloodstream, hydroxylated-PMFs are better absorbed through oral consumption. Nonetheless, both have shown diverse health benefits, e.g., the regulation of lipid metabolism, anti-diabetes, anti-obesity, anti-inflammation, and anti-cancer [27].

### 2.2. Evolution of Major Metabolites in Growing Fruits during the First Weeks of Growth

To analyze the influence of fruit size on the metabolites produced, during the first weeks of growth, raw data were separated according to variety for data processing and statistical analyses. After the compound detection, QC correction, and processes, the four matrices (for each variety) containing 193 RT-*m*/*z* variables each, along with their corresponding normalized areas, were utilized for statistical analyses.

**Figure 4:**
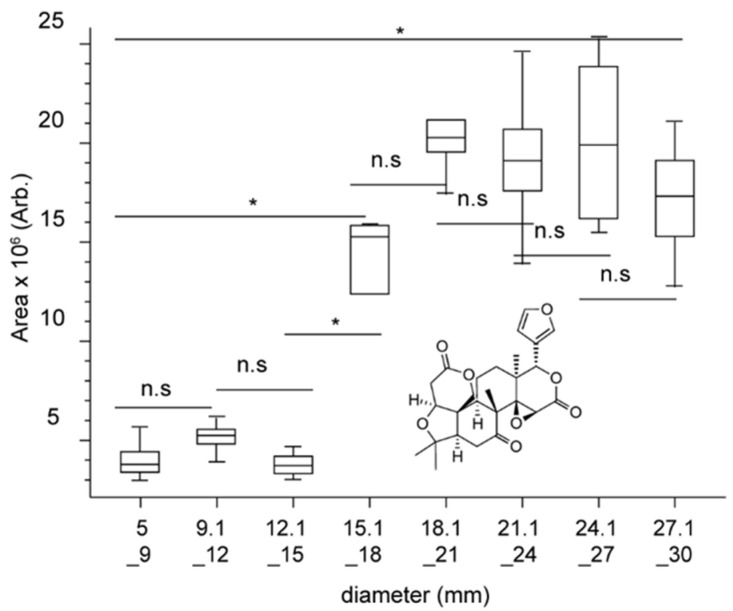
Box-plot comparison of the intensities of limonin according to the size of Fremont mandarins. * log(fold change) > 1.0 and *p*-value < 0.05; n.s.: statistically non-significant difference.

A first PLS-DA was performed using the fruit size for the model. However, the model had low goodness of fit (0.418 < R2X < 0.555; 0.209 < R2Y < 0.262) and very low predictability (0.087 < Q2Y < 0.157) values. Manual inspection of the data revealed that for many compounds, the increase/decrease in intensity from one size to the next was often too small to be statistically significant. Moreover, for most metabolites, a strong increase/decrease around the 18 mm fruits was observed, potentially coinciding with half the duration of the collection period. Hence, the differences in intensity between the smallest size (5–9 mm), the middle size (15.1–18 mm and 18.1–21 mm), or the largest size (27.1–30 mm) were statistically significant (Figure 4).

The development of citrus fruits occurs in three distinct phases: cell division, lasting about 8 weeks after flowering; cell enlargement, spanning 3 to 6 months; and ripening. Each phase is marked by specific changes in primary and secondary metabolisms [28,29]. The early stages are crucial for the accumulation of secondary metabolites, including phenolic compounds and flavonoids. Notably, the accumulation of organic acids peaks in the middle of the cell enlargement phase, then gradually decreases, while sugars continue to accumulate, reaching their maximum at the end of maturation [5]. These results clearly highlight the metabolic differences between the first two phases, allowing us to categorize the samples into two distinct size groups, (i) from 5 mm to 18 mm and (ii) from 18.1 mm to 30 mm, which agrees with the PLS-DA models acceptable for all varieties in this configuration (0.405 < R2X < 0.541; 0.844 < R2Y < 0.953; 0.718 < Q2Y < 0.908). In addition, volcano plots were generated by comparing each size to the next, size 5–9 mm against 15.1–18 mm, size 18.1–21 mm against 27.1–30 mm, and size 5–9 mm against 27.1–30 mm. Variables with log(fold change) > 1.0 and *p*-value < 0.05 for at least three of the comparisons were kept.

Seventy-two variables were selected as significantly different (VIP > 1.0; log(fold change) > 1.0; *p*-value < 0.05), and amongst them, five were identified by the corresponding standard (fructose, adenosine, 5-demethylnobiletin, isonaringin, and neoponcirin), sixteen were tentatively identified by correspondence with MS/MS databases, and fifty-one were annotated as isomers of identified compounds or by their chemical family. Details of the annotations are given in Table S2 of the Supplementary Materials.

The compounds identified could be divided into two groups depending on their intensity evolution by either an (i) increase (40) or (ii) decrease (31) with the growth of the fruit. The distribution of the identified compounds per chemical family from groups (i) and (ii) is given in Table 2.

Almost all compounds had a similar evolution in all varieties except for one compound, a sugar with the molecular formula C_11_H_16_O_13_ (RT: 4.2 min, *m*/*z* 357.0662), for which a significant increase was observed for the ‘Navel’ orange, while a significant decrease was observed for the ‘Valencia’ orange (non-significant evolution for ‘Fremont’ mandarin and ‘Valencia Late’ orange).

**Figure 5:**
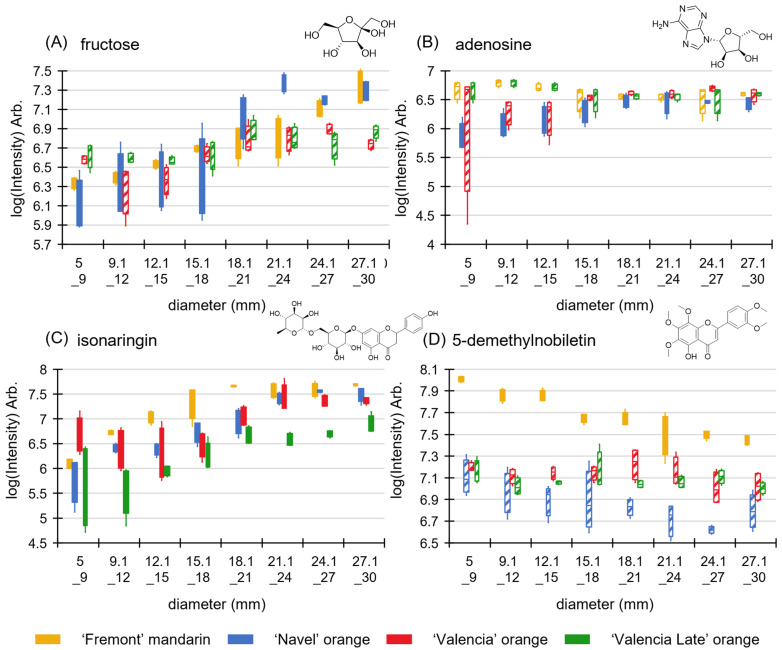
Box-plot comparison of intensities by variety and size for (**A**) fructose, (**B**) adenosine, (**C**) isonaringine, and (**D**) 5-demethylnobiletine. Orange: ‘Fremont’ mandarin, blue: ‘Navel’ orange, red: ‘Valencia’ orange, green: ‘Valencia Late’ orange; crosshatching: non-significant change according to established criteria.

Figure 5 illustrates these results for four compounds identified by confirmation with a standard (increase for (A) fructose, (B) adenosine, and (C) isonaringin; decrease for (D) 5-demethylnobiletin). Only isonaringin and an amino sugar (RT 2.5 min, *m*/*z* 295.1133) demonstrated significant changes across all four varieties, whereas for the other compounds, there was no significant evolution in at least one variety, as illustrated in Figure 5 for fructose, adenosine, and 5-demethynobiletin. Therefore, these results imply that the evolution of metabolite level with the fruit size varies depending on the variety or cultivar, even during the first weeks of growth. Indeed, similar observations were made by Multari et al. [22] and Bi et al. [14], albeit in relation to more mature fruits.

While there have been numerous publications on the metabolites associated with citrus growth, there is a notable lack of literature dealing with the smallest fruit sizes. As most citrus trees begin flowering in spring [30], citrus samples collected in September [22,31] or later [32] are more advanced in their growth than those used in this study or may correspond to the largest fruit size [13,15,24,33]. Still, our findings could be compared to the study of Goh et al. [26] on the development of metabolites in the peel and juice of ‘Navel’ oranges over time. Their study started in the seventh week, which could potentially correspond to the midpoints of our examination. The second sample was collected in the 20th week, exceeding the estimated growth duration for the 30 mm ‘Navel’ orange fruits studied (8 to 9 weeks). The decline in neoponcirin and limonin with fruit development was consistent across both studies. However, some compounds (e.g., tangeretin, sinensetin) underwent significant changes in their research, which were not reflected in our findings due to the relatively minor alterations in content that we observed (i.e., log(fold change) < 1). Interestingly, opposite trends were observed for isonaringin, with a decline noted during fruit development reported by Goh et al. [26], which was in accordance with the findings of Kumar et al. [18].

These discrepancies might be due to the studied cultivar [34], fruit drying conditions (e.g., sun-dried, hot-air-dried, or freeze-dried) [18,31], and/or geographical origins [35].

Finally, it should be noted that there was a substantial fluctuation in the levels of several metabolites in the 18 mm fruits —i.e., during weeks 7 or 8 depending on the variety, with either a significant increase or decrease. Therefore, our findings indicate that including the smallest-sized fruits can provide new insights in the study of citrus growth.

## 3. Materials and Methods

### 3.1. Chemicals

Methanol, acetonitrile, and water were purchased from Fischer Chemical (Loughborough, UK), whereas formic acid was from Merck (Darmstadt, Germany) and dimethyl sulfoxide (DMSO) was from Thermo Scientific (Waltham, MA, USA). All solvents and formic acid were LC-MS grade. All standards were analytical standards with a purity ≥ 95%. Sinensetin, tangeretin, 5-demethylnobiletin, nobiletin, and gardenin A were purchased from Biosynth (Compton, UK). Fructose, adenosine, isonarigin, and neoponcirin were purchased from Sigma Aldrich (Steinheim, Germany). 3′,4′,5,7-tetramethoxyflavone was purchased from Thermo Scientific (Altrincham, UK).

### 3.2. Fruit Samples and Sample Preparation

Small fruits were collected from the south side of adult trees grown in an experimental orchard near San Giuliano in Corsica (42°18′55″ N, 9°29′29″ E; 51 m above sea level). Trees were from the collection of the “CRB Citrus” biological resource center managed by INRAE and CIRAD in Corsica (France). The trees were submitted to standard cultural practices: water was supplied every day on the basis of the 100% replacement of actual evapotranspiration estimated from the Penman–Monteith equation [36] and fertilizers were supplied according to the recommendations of the local agriculture department. Insects and diseases were also controlled according to these recommendations. Three cultivars of orange (*Citrus sinensis* (L.) Obs.) and one of mandarin (*Citrus reticulata* Ten.) were analyzed (Table 3).

The experiments were conducted in open fields and standard procedures were applied to minimize variations due to potentially different environmental conditions, as previously reported [37]. For each variety, 4 genetically identical trees (grafted on the same rootstock) were used as replicas. To follow the evolution of metabolites during fruit development, a minimum of 100 g of fresh fruit and at least 10 fruits were collected and classified into 8 groups, according to the following sizes of fresh fruit: 5–9 mm, 9.1–12 mm, 12.1–15 mm, 15.1–18 mm, 18.1–21 mm, 21.1–24 mm, 24.1–27 mm, and 27–30 mm. In Corsica, citrus flowering is spread over 4–6 weeks, depending on the spring climate. Since citrus flowers do not bloom simultaneously, different fruit sizes can coexist, especially in the early stage of the growth. Therefore, all sizes were harvested simultaneously on 28 June 2022.

After being harvested, measured, and weighed, the fruits were dried in an oven (Memmert Elektronik BE400 220 V 1000 W 70°, Bioblock Scientific, Madrid, Spain) at a temperature of 50 °C for 3 weeks in order to achieve complete desiccation while preserving the most bioactive compounds [31]. Then, the dried fruits were crushed with a blade crusher (Moulinex Grinder AR-1108 180W, Moulinex, Paris, France) to a fineness of 400 μm.

For the LC-MS analysis, citrus powders were diluted in a 1:1 water/methanol mixture (2 mg/mL), filtered through 13 mm GHP 20 µm filters (acrodisc), and then diluted 10-fold in a 1:1 water/methanol mixture. Standards were prepared in DMSO (1 mg/mL) and diluted 50-fold in a 1:1 water/methanol mixture. Previous experiments (Supplementary Materials) demonstrated that the experimental deviation was negligible in comparison with the biological variation. Consequently, no extraction replicates were conducted. In total, four replicates (biological replicates) per condition (variety and size) were employed.

### 3.3. Instrumentation

The LC-MS/MS experiments were performed using an ultra-high performance liquid chromatography (UHPLC) system (Vanquish, Thermo Scientific, San Jose, CA, USA) coupled to a hybrid quadrupole–Orbitrap mass spectrometer (Orbitrap Exploris 120, Thermo Scientific, San Jose, CA, USA) using heated electrospray ionization (HESI). The acquisition was performed using Xcalibur software 4.1 (Thermo Scientific, San Jose, CA, USA). The chromatographic gradient and mass spectrometer parameters were optimized prior to the analysis. For instance, the optimization allowed us to minimize in-source fragmentation.

The column was a 2.1 mm × 100 mm Acquity UPLC CSH C18 1.8 µm column (Waters, Manchester, UK) with a 0.2 µm pre-filter. The sample tray and column oven temperatures were set at 20 °C and 40 °C, respectively. The injection volume was set at 1 µL. A flow rate of 100 μL/min was used. Mobile phase A consisted of water + 0.1% formic acid and mobile phase B of acetonitrile + 0.1% formic acid. The gradient elution was as described in Table 4.

Before and after each run, an experimental blank was injected for blank subtraction. The column was equilibrated with four injections of the quality control (QC, mix of every samples), and then, the QC was injected throughout the runs after every eight samples. Samples were randomly injected as recommended for metabolomic experiments [19].

Mass spectra were obtained in the positive ionization mode. The source parameters were set as the following: The spray voltage, ion transfer tube temperature, and vaporizer temperature were 3.1 kV, 300 °C, and 400 °C, respectively. Sheath gas, auxiliary gas, and purge gas were set at 45 units, 20 units, and 5 units, respectively. Mass spectra were recorded over a *m*/*z* 150–1500 range using full scan and data-dependent acquisition (ddMS^2^) modes with a resolution setting of 120,000 FWHM for the MS^1^ and 15,000 for the MS^2^. For the ddMS^2^, the intensity threshold and normalized collision energy were set up to 9 × 10^4^ and 22%, respectively. Dynamic exclusion was performed by excluding ions that were selected twice within 1 s for 3 s.

### 3.4. Data Processing and Data Analysis

#### 3.4.1. Data Processing

Data processing (compound detection and normalization) was performed using Compound Discoverer 3.3.2.31 software (Thermo Fisher Scientific). The workflow and parameters are given in Table S1 of the Supplementary Materials. Matrices containing RT-*m*/*z* variables, along with their corresponding normalized areas, were exported from the software for the PLS-DA. Box-plot graphs were also exported from Compound Discoverer.

#### 3.4.2. Statistical Analysis

The log(fold change) and *p*-values resulting from the different comparisons (e.g., ‘Fremont’ mandarin vs. ‘Valencia’ orange with all sizes included) were calculated by the Compound Discoverer 3.3.2.31 software (Thermo Fisher Scientific) and exported in .csv format for further analysis. PLS-DA were performed by importing the data matrices processed by Compound Discoverer on the Workflow4Metabolomics.org (W4M) platform [38,39]. The VIP score resulting from the PLS-DA was exported from the W4M platform in .csv format. Subsequently, the two files were manually merged using Excel for Microsoft 365 MSO (Microsoft), with significantly different variables retained if they satisfied all the specified criteria (VIP > 1.0; log(fold change) > 1.0; *p*-value < 0.05). Score plots from the PLS-DA were generated by the W4M platform, and chemical structures were drawn using Chemdraw Ultra 12.0 (CambridgeSoft, Cambridge, MA, USA).

#### 3.4.3. Metabolite Identifications

Identification was performed on MS/MS spectra and confirmed by standards when available. The annotation of compounds was then classified according to the annotation levels of Schymanski et al. [20]: structural identification verified by using a standard (identical retention time (RT), molecular formula, and MS/MS spectrum); 2: structural identification based on similarity with an internal or external database (molecular formula and MS/MS spectrum); 3: chemical family identification (molecular formula and MS/MS spectrum); 4: molecular formula; and 5: RT and *m*/*z*. The MS/MS databases used were mzCloud (https://www.mzcloud.org/, accessed on 12 February 2024, Thermo Fisher Scientific), the Mass Bank of North America (MoNA, http://mona.fiehnlab.ucdavis.edu, accessed on 12 February 2024), the Global Natural Product Social Molecular Networking (GNPS, http://gnps.ucsd.edu, accessed on 12 February 2024), and the Human Metabolome Database (HMBD, http://www.hmdb.ca/, accessed on 12 February 2024). The literature was also used for MS/MS spectra obtained solely from standards [23,40,41,42,43,44,45].

## 4. Conclusions

In conclusion, this study provides insight into the metabolite composition of four common citrus varieties in their first weeks of growth, notably in the determination of metabolic variations among their varieties or depending on the fruit size. Fifty-six metabolites were determined as significantly different between the immature mandarin and the three immature orange cultivars, of which thirty-five, more than half, were secondary metabolites of the polyphenol family, molecules highly sought after for their health benefits.

Additionally, 21 metabolites were determined as significantly different between the immature ‘Navel’ orange and the immature ‘Valencia’ orange or immature ‘Valencia Late’ orange. Given that oranges are genetically almost identical, with only minor point mutations distinguishing them, this method could serve as a preliminary approach for the identification of different orange varieties based on their accumulation of primary and secondary metabolites.

The analysis of the influence of the fruit diameter on the four citrus cultivars demonstrated, in correlation with the bibliography, a distribution of samples into two distinct groups: from 5 mm to 18 mm and from 18.1 mm to 30 mm diameters. This shows that increasing fruit size resulted in a significant increase for 40 metabolites and a significant decrease for 31 metabolites.

While it was not feasible to strictly identify all compounds due to the absence of available standards, each compound was labeled with the respective chemical family. As such, beyond the typical focus in citrus studies on flavonoids, limonoids, and sugars, this investigation uncovered other classes such as amino acids, amino sugars, and lipids. It is worth mentioning that a high number of polymethoxyflavone compounds have been reported, including numerous isomers, i.e., four isomers of demethylnobiletin. As polymethoxyflavones have been associated with many health benefits, this study in its entirety (interspecific, different phases of development) highlights the relevance of immature citruses for the pharmaceutical industry, particularly with regard to the 18 mm stage, which corresponds to 7/8 weeks after flowering, i.e., the transition between the end of the cell division phase and the cell enlargement phase.

In view of current climate change, a valuable addition to this research would be to extend its geographical scope to a multiregional level, thus gaining deeper insights into the metabolic responses of different species during the initial stages of citrus fruit development.

## Figures and Tables

**Table 1:** Distribution of the identified compounds per chemical family that were either up-regulated in the ‘Fremont’ mandarin or the three oranges.

Chemical Family	Number of Up-Regulated Compounds in the ‘Fremont’ Mandarin	Number of Up-Regulated Compounds in the Oranges
Amino acids	0	4
Amines	4	3
Peptides	0	1
Amino sugars	0	4
Sugars	0	2
Lipids	1	1
Polyphenols	0	6
Polymethoxyflavones	11	10
Polymethoxyflavanones	0	1
Flavones	2	5
Organic phosphorous	0	1

**Table 2:** Distribution of the identified compounds per chemical family that either showed an intensity increase or decrease with the fruit growth.

Chemical Family	Number of Compounds with an Intensity Increase	Number of Compounds with an Intensity Decrease
Organic acids	1	0
Amino acids	1	0
Peptides	0	1
Amines	5	11
Amino sugars	10	1
Sugars	4	2
Lipids	0	3
Polyphenols	3	3
Limonoids	3	0
Polymethoxyflavones	3	10
Flavones	3	0
Flavanones	2	0
Organic phosphorus	5	0

**Table 3:** Cultivar and rootstock combinations analyzed in this study.

Variety	*Citrus sinensis* (L.) Obs.	*Citrus sinensis* (L.) Obs.	*Citrus sinensis* (L.) Obs.	*Citrus reticulata* Ten.
Cultivar	Valencia campbell	Valencia Late	Navelate	Fremont
	SRA 17	SRA 246	SRA 307	SRA 147
Rootstock	*Poncirus trifoliata*	*Poncirus trifoliata*	*Poncirus trifoliata*	*Poncirus trifoliata*
Plot location	Ala H1-4	Ala H1-4	Ala O17-20	B3 G1-4

**Table 4:** Chromatographic gradient settings.

Time (min)	Mobile Phase B (%)
0	0
2	0
4	40
10	100
14	100
14.5	0
16	0

## Data Availability

Data are available on request due to restrictions, i.e., privacy. The data presented in this study are available on request from the corresponding author.

## Supplementary Materials

The following supporting information can be downloaded at https://www.mdpi.com/article/10.3390/molecules29163718/s1: Figure S1: Verification of the extraction variability; Figures S2–S4: Illustration of compound annotation according to the first 3 levels of annotation confidence of Schymanski et al. [20]; Table S1: Metabolomic workflow and parameters on Compound Discoverer software; Table S2: Summary of the identified metabolites according to differences between (i) the three oranges and the Fremont mandarin, (ii) the Navel orange and Valencia/Valencia Late oranges, or that increases or decreases with the fruit growth, with MS/MS spectra available. Table S3: Evolution. The references used for the identification are given in Section 3.4.3.
