# Supplementary material for: Metabolite Variations during the First Weeks of Growth of Immature Citrus sinensis and Citrus reticulata by Untargeted Liquid Chromatography–Mass Spectrometry/Mass Spectrometry Metabolomics
Source: Molecules. 2024 Aug 6;29(16):3718. doi: 10.3390/molecules29163718 (PMC11357260; doi:10.3390/molecules29163718)
Supplement: Supplementary file 1 [file molecules-29-03718-s001.zip › S1_Deschamps_Molecules.pdf]

Metabolite Variations during the First Weeks of Growth of Immature *Citrus sinensis* and *Citrus reticulata* by Untargeted Liquid Chromatography–Mass Spectrometry/Mass Spectrometry Metabolomics

Estelle Deschamps [1], Marie Durand-Hulak [2,3], Denis Castagnos [4], Marie Hubert-Roux [1], Isabelle Schmitz [1,*,†], Yann Froelicher [3] and Carlos Afonso [1,*]

1 Institut National des Sciences Appliquées (INSA) Rouen Normandie, Univ Rouen Normandie, Centre National de la Recherche Scientifique (CNRS), Normandie Univ, Chimie Organique et Bioorganique Ré-activité et Analyse (COBRA) UMR 6014, INC3M FR 3038, 76000 Rouen, France; es-telle.deschamps1@univ-rouen.fr (E.D.); marie.hubert@univ-rouen.fr (M.H.-R.)

2 EARL DURAND Olivier, Domaine de la Triballe, 34820 Guzargues, France; marie-durand@live.fr

3 Centre de Coopération Internationale en Recherche Agronomique pour le Développement (CIRAD), UMR AGAP Institut, Station INRAE, 20230 San Giuliano, France; yann.froelicher@inrae.fr

4 ORIL Industrie, Servier Group, 13 r Auguste Desgenétais, 76210 Bolbec, France; de-nis.castagnos@servier.com

* Correspondence: isabelle.schmitz@cnrs.fr (I.S.); carlos.afonso@univ-rouen.fr (C.A.)

† Current address: Univ Rouen Normandie, INSA Rouen Normandie, CNRS, Normandie Univ, Polymères Biopolymères Surfaces (PBS) UMR 6270, 76000 Rouen, France.

Supporting information list

- Table S1 Metabolomic workflow and parameters on Compound Discoverer software
- Figure S1 Verification of the extraction variability (unpublished previous work)
- Figures S2–4 Illustration of compound annotation according to the first 3 levels of annotation confidence of Schymanski *et al.*[1]
- Table S2 Summary of the identified metabolites according to differences between i) the three oranges and the *Fremont* mandarin, ii) the *Navel* orange and *Valencia* / *Valencia Late* oranges, or that increases or decreases with the fruit growth, with MS/MS spectra available

[1] Schymanski, E. L., Identifying Small Molecules via High Resolution Mass Spectrometry: Communicating Confidence, *Environ. Sci. Technol. 2014*, 48, 2097−2098

LC-MS/MS data were processed with Compound Discoverer 3.3.2.31 software (Thermo Fisher Scientific) using the workflow and parameters in Table S1 (if not indicated, parameters recommended by the software). The following study factors were used: variety (*Fremont* mandarin, *Navel* orange, *Valencia* orange, and *Valencia Late* orange), diameter (05_9, 09.1_12, 12.1_15, 15.1_18, 18.1_21, 21.1_24, 24.1_27, and 27.1_30), and group (5_9, and 18.1_30). Molecular formulae were manually checked and corrected from the raw data using Freestyle 1.8.51.0 software (Thermo Fisher Scientific).

Table S1 Metabolomic workflow and parameters on Compound Discoverer software

Step	Parameter	Value
1 Select spectra	Upper RT limit (min)	11
2 Align retention times (ChromAlign)	Reference file	QC
3 Detect compounds	Mass tolerance (ppm)	3
	Min. peak Intensity	200 000
	ions	$[2M+H]^+$, $[2M+Na]^+$, $[M+H]^+$, $[M+Na]^+$, $[M+NH_4]^+$
	Base ions	$[M+H]^+$, $[M+Na]^+$
4 Group compounds	Mass tolerance (ppm)	3
	RT tolerance (min)	0.2
	Preferred ions	$[M+H]^+$, $[M+Na]^+$
	Peak rating threshold	3
	Number of files	4
5 Fill gaps	Mass tolerance (ppm)	3
	S/N threshold	1.5
6 Apply SERRF QC correction	Min. QC coverage (%)	25
	Max. QC area RSD (%)	50
	# batches	1
7 Normalize area	Normalization type	constant sum
8 Mark background compounds	Max. sample/blank	5
9 Predict compositions	Mass tolerance (ppm)	3
	Min. element counts	C H
	Max. element counts	C40 H80 N9 O30
	Min. H/C	0.7
	Max. H/C	2.8
	Use fragment matching	true
	Mass tolerance (ppm)	5
	S/N threshold	3
10 Search mzCloud	Compound classes	endogenous metabolites, natural products/ medicines
	Precursor mass tolerance (ppm)	5
11 Assign compound annotations	Mass tolerance (ppm)	3
	data source #1	Predicted compositions
	data source #2	mzCloud search
12 Post-processing nodes: Differential analysis	log10 transform value	true

In a previous experiment, the variability of the extraction was tested on a smaller number of samples. The extraction process was conducted in triplicate on three separate days. The subsequent score plot derived from the principal component analysis (PCA) illustrates that the variability associated with the extraction procedure is sufficiently low to permit the assignment of biological differences (Figure S1).

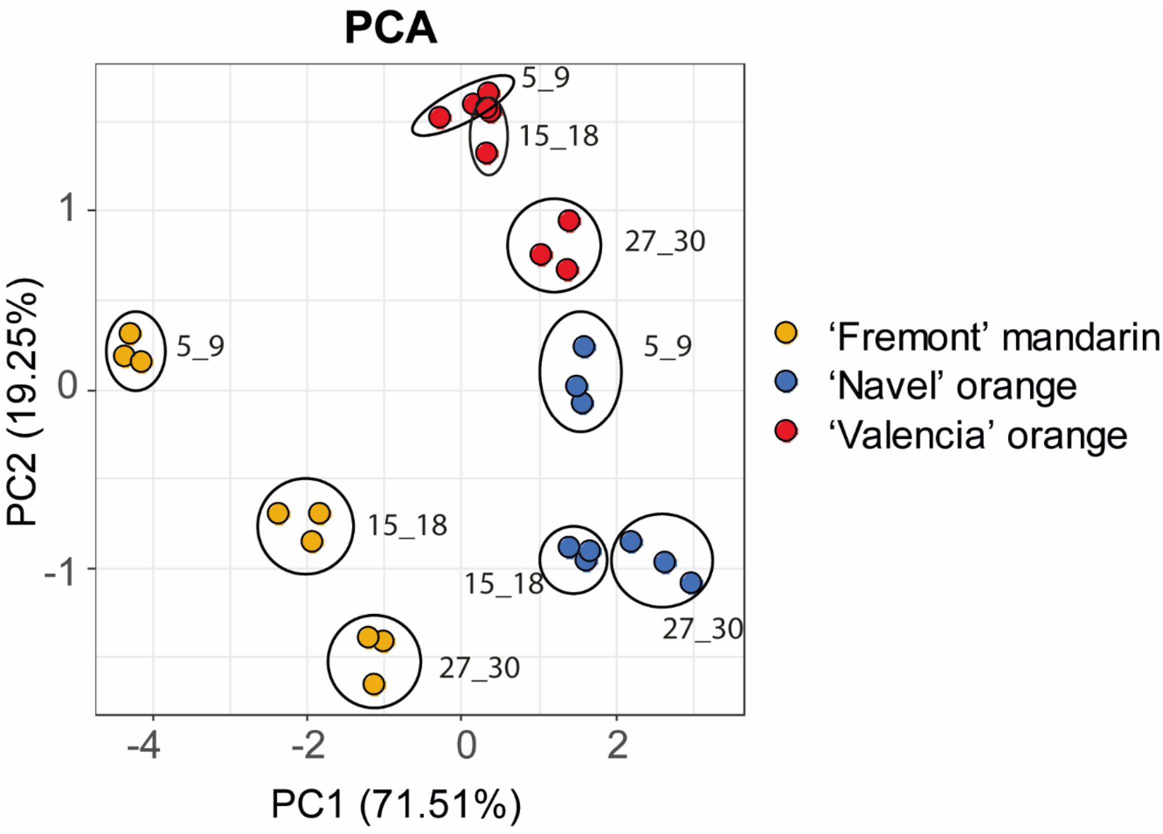

Figure S1. Score plot obtained from the PCA performed on extraction triplicates. Orange: 'Fremont' mandarin; blue: 'Navel' orange; red: 'Valencia' orange. Fruit sizes (5_9 mm, 15_18 mm, 27_30 mm) are indicated by circles.

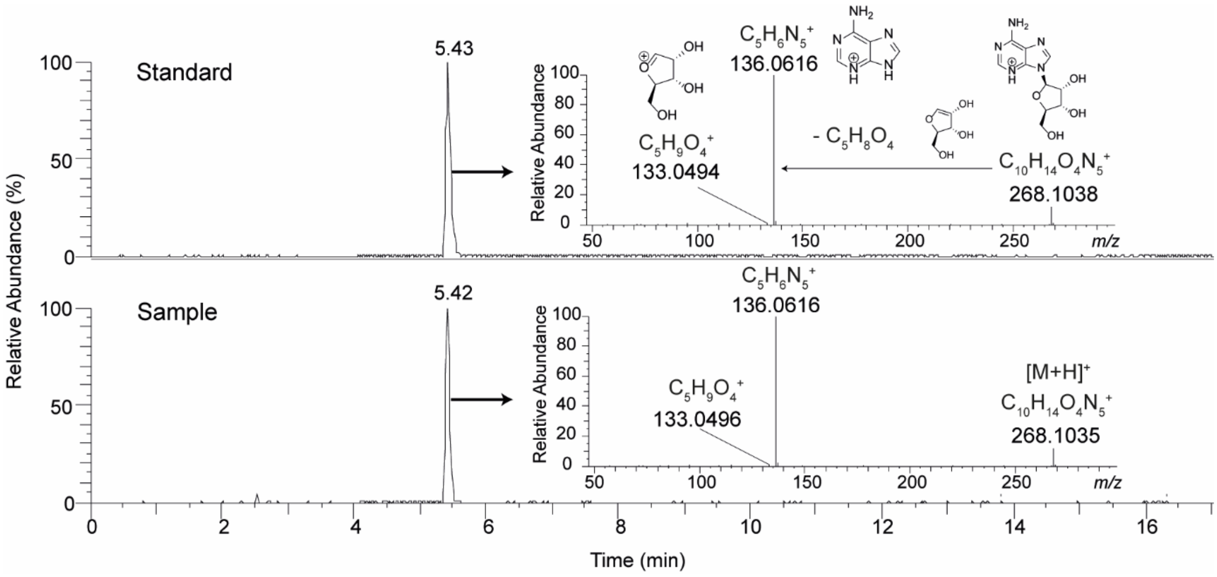

Figure S2. Example of the level 1 identification of adenosine: retention time, molecular formula and MS/MS fragments matched exactly between the sample and the standard.

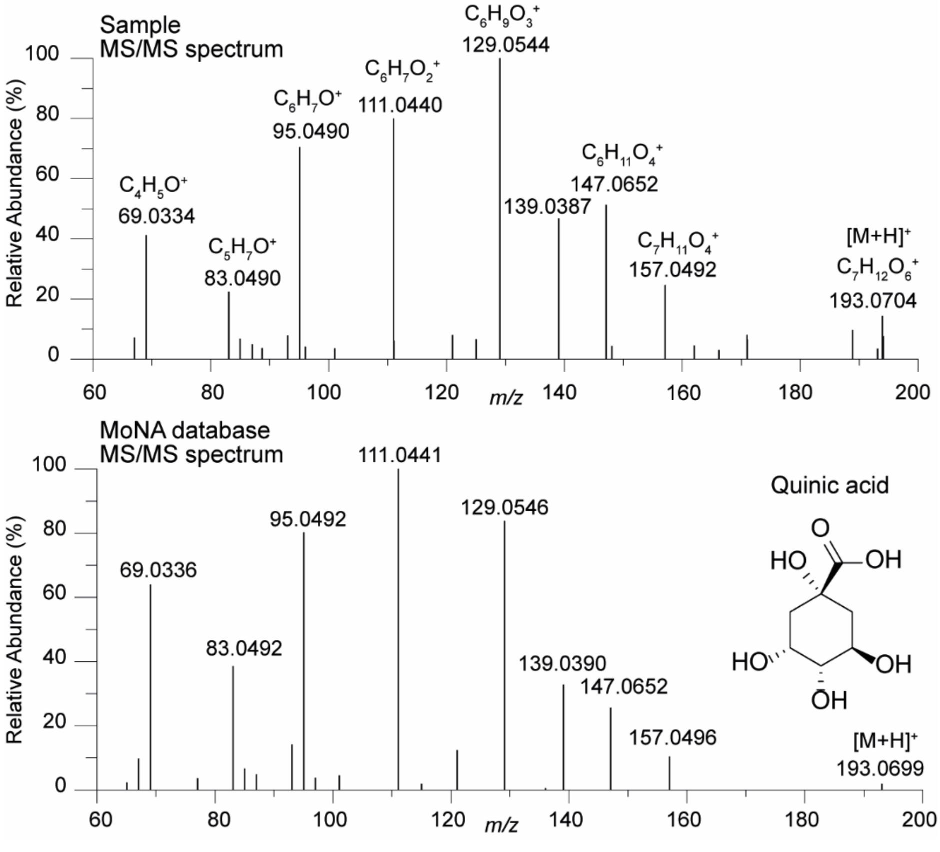

Figure S3. Example of the level 2 identification of quinic acid: molecular formula and MS/MS fragments matched between the sample and the database (MoNA).

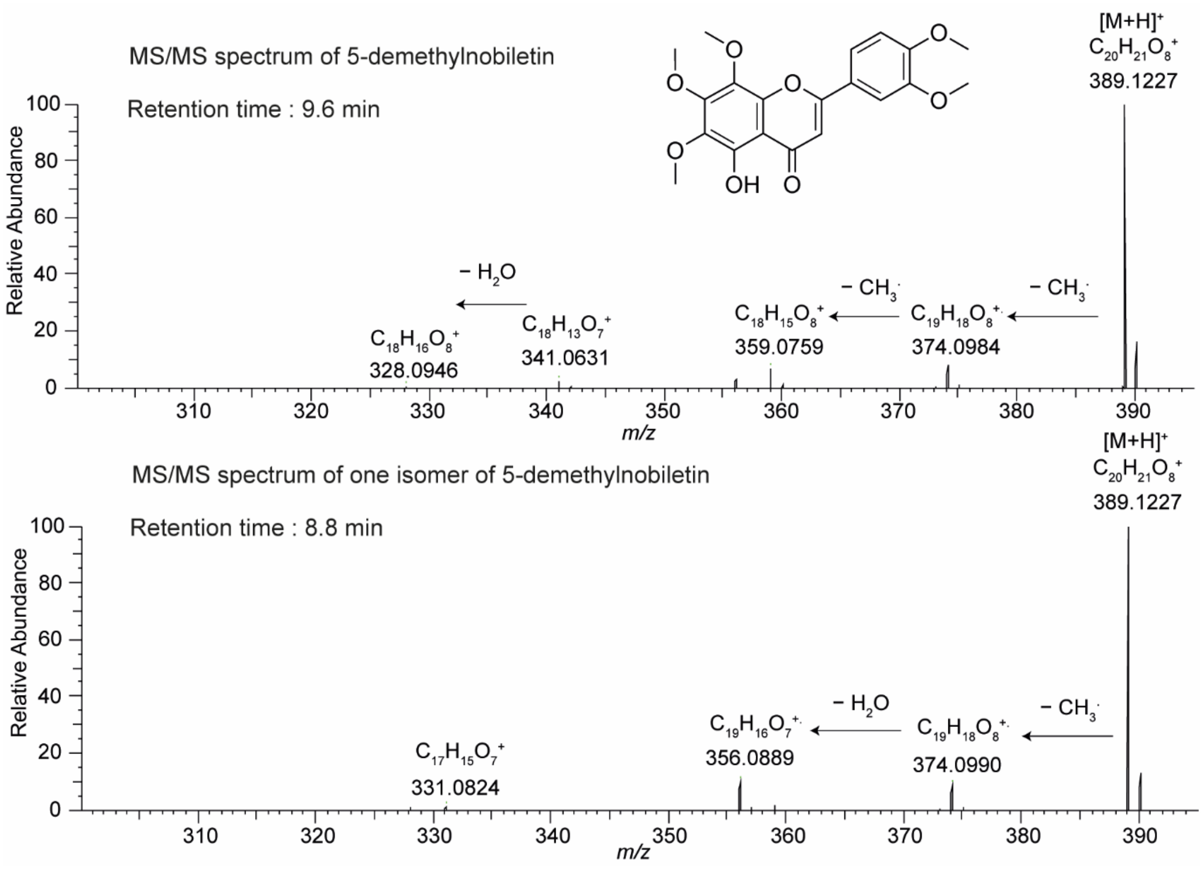

Figure S4. Example of the level 3 identification of the isomer of 5-demethylnobiletin. In this example, although no match was found in the literature for the *m/z* 389.1227 eluting at 8.8 min, the fragmentation similarity with 5-demethylnobiletin allowed to assign its chemical family of as polymethoxyflavones.
